# Genome-Wide Analysis of *R2R3-MYB* Genes and Functional Characterization of *SmMYB75* in Eggplant Fruit Implications for Crop Improvement and Nutritional Enhancement

**DOI:** 10.3390/ijms25021163

**Published:** 2024-01-18

**Authors:** Suli Shi, Dalu Li, Shaohang Li, Na Zhao, Jielei Liao, Haiyan Ge, Yang Liu, Huoying Chen

**Affiliations:** School of Agriculture and Biology, Shanghai Jiao Tong University, Shanghai 200240, China

**Keywords:** *R2R3-MYB* genes, genome-wide characterization, eggplant, *SmMYB75*, metabolite

## Abstract

*R2R3-MYB* represents a substantial gene family that plays diverse roles in plant development. In this study, 102 *SmR2R3-MYB* genes were identified from eggplant fruit and classified into 31 subfamilies. Analysis indicated that segmental duplication events played a pivotal role in the expansion of the *SmR2R3-MYB* gene family. Furthermore, the prediction of miRNAs targeting *SmR2R3-MYB* genes revealed that 60 *SmR2R3-MYBs* are targeted by 57 miRNAs, with specific miRNAs displaying varying numbers of target genes, providing valuable insights into the regulatory functions of miRNAs in plant growth, development, and responses to stress conditions. Through expression profile analysis under various treatment conditions, including low temperature (4 °C), plant hormone (ABA, Abscisic acid), and drought stress (PEG, Polyethylene glycol), diverse and complex regulatory mechanisms governing *SmR2R3-MYB* gene expression were elucidated. Notably, *EGP21875.1* and *EGP21874.1* exhibited upregulation in expression under all treatment conditions. Transcriptome and metabolome analyses demonstrated that, apart from anthocyanins (delphinidin-3-O-glucoside, cyanidin-3-O-(6-O-p-coumaroyl)-glucoside, and malvidin-3-O-(6-O-p-coumaroyl)-glucoside), overexpression of *SmMYB75* could also elevate the content of various beneficial compounds, such as flavonoids, phenolic acids, and terpenes, in eggplant pulp. This comprehensive study enhances our understanding of *SmR2R3-MYB* gene functions and provides a strong basis for further research on their roles in regulating anthocyanin synthesis and improving eggplant fruit quality.

## 1. Introduction

Transcription factors (TFs) play vital roles in the regulation of plant growth and development as well as metabolic processes by controlling the transcription levels of structural genes in various biological processes [1]. The MYB (V-myb avian myeloblastosis viral oncogene homolog) TFs represent one of the largest plant transcription factor families, containing highly conserved MYB DNA-binding domains widely distributed throughout eukaryotic organisms. The MYB gene family has been thoroughly identified and functionally studied in an increasing number of species over the past few decades [2,3,4]. The structural domains of the genes in the MYB family typically consist of one to four non-identical repeats called R repeats (R1, R2, R3, and R4), each approximately 50–55 amino acids in length [5]. The genes can be divided into four subfamilies based on the number of R repeats: 1R-MYB (MYB-related and R3-MYB), R2R3-MYB factors, 3R-MYB (R1R2R3-MYB), and 4R-MYB. The R2R3-MYB factors are the most abundant and most common in plants and regulate various aspects of plant growth and development [6], primary and secondary metabolism [7,8,9], biotic and abiotic stress responses [10,11,12,13], and cell morphology development [14].

The R2R3-MYB TFs play key roles in the regulation of flavonoid biosynthesis in various species. Anthocyanins, secondary metabolites of flavonoids, are the primary pigments responsible for the red, purple, and blue hues in plants, aid in biotic and abiotic stress resistance, and have also been used in cancer therapy [15]. The anthocyanin synthesis pathway is a branch of the flavonoid synthesis pathway and involves a variety of key gene-encoding enzymes. It involves the early biosynthetic genes (EBG; such as *CHS*, *CHI*, and *F3H*), late biosynthetic genes (LBG; such as *DFR*, *ANS*, and *BZ1*, which encode the UDP glucose-like flavonoid glycosyltransferase (UFGT)), and other components [16,17,18]. The R2R3-MYB proteins often form MYB-bHLH-WD40 complexes with bHLH and WD40 proteins to regulate anthocyanin biosynthesis. However, the R2R3-MYB proteins can also act independently as activators to control structural gene expression involved in anthocyanin biosynthesis [19,20].

The first plant-specific *R2R3-MYB* gene *ZmMYBC1* identified in maize (*Zea mays*) encodes a regulatory protein involved in anthocyanin biosynthesis [21,22]. Furthermore, the R2R3-MYB-mediated anthocyanin synthesis pathway was discovered in the model *Arabidopsis*, where *AtPAP1/AtMYB75* and *AtPAP2/AtMYB90* were shown to be involved in the transcriptional regulation of anthocyanins in plant tissues [23,24]. In tomatoes, the overexpression of *SlANT1, SlANT2/SlMYB75*, and *SlANT2-like R2R3-MYB* genes increases fruit anthocyanin content [25]. Both MdMYB9 and MdMYB11 in apples bind to the promoters of the structural anthocyanin synthesis genes *ANS, ANR*, and *LAR*, activating their expression and resulting in increased anthocyanin accumulation [26].

Eggplant pericarp is rich in anthocyanins, which is a significant factor in the quality of eggplant fruits [27]. However, since only the eggplant peel is enriched in anthocyanins but not in the flesh, the overall nutritional value of eggplant is less valued. A previous study demonstrated that *SlMYB75* overexpression was effective in improving various fruit quality characteristics, including a significant increase in total phenolics, flavonoids, soluble solids, and anthocyanins in *SlMYB75-OE* fruits. Flavonoids are a group of polyphenolic hydroxyl compounds widely distributed in the plant kingdom, most of which are yellow in color and have various biological functions such as anti-free radicals, antioxidants, immune enhancement, and anti-aging [28]. Therefore, fruit quality could be improved to some extent if significant positive regulators can be identified in eggplant fruit. Previous reports have shown that anthocyanin synthesis in eggplant pericarp is light-dependent with structural genes (such as *SmCHS*, *SmCHI*, *SmF3H*, *SmDFR*, and *SmANS*) cloned and their functions elucidated [29,30]. In recent years, several *R2R3-MYB* genes involved in anthocyanin biosynthesis have been identified in eggplant fruit. *SmMYB* was first cloned from the purple petals of eggplant fruit and the expression trend in the pericarp was shown to be positively correlated with changes in anthocyanin content [31]. Furthermore, *SmMYB1* [32], *SmMYB75* [33], and *SmMYB35* [34] were shown to promote anthocyanin synthesis and *SmMYB86* was shown to be a negative regulator of anthocyanin biosynthesis [35]. In recent years, with the development of genome sequencing, many genomes have been published and genome-wide identification of large gene families (i.e., *R2R3-MYB*) have been completed, including those in *Arabidopsis* [36], tomato [37], pepper [38], potato [39], and tobacco [40] plants, among others. However, the current reports on the *R2R3-MYB* gene family in eggplant fruit are relatively sparse with only a few genes being functionally verified.

In this study, a comprehensive study of the *R2R3-MYB* gene family in eggplant fruit was conducted using genomic sequence data reported by Guangxi University [41]. The analysis included gene structure, chromosomal location, phylogenetic relationships, pattern composition, duplication events, and predicted cis-acting elements. In addition, the differential expression levels of *SmR2R3-MYBs* in response to abiotic stresses were analyzed. Overexpression of the *SmMYB75* gene produced eggplants with purple flesh and further revealed its regulatory role in eggplant fruit quality via transcriptomics and metabolomics. Completely purple eggplants can be commercially favored and are somehow considered better quality eggplants. These findings provide a putative strategy for anthocyanin-rich eggplant cultivar breeding.

## 2. Results

### 2.1. Identification and Physicochemical Property Analysis of the SmR2R3-MYB Genes

A BLAST query of *Arabidopsis AtR2R3-MYBs* was performed against the eggplant genome to identify the putative *SmR2R3-MYBs* in eggplant. A total of 102 candidate genes were identified using the SMART (http://smart.embl-heidelberg.de/, accessed on 18 September 2023) and Pfam databases (https://www.uniprot.org/database/DB-0073, accessed on 18 September 2023) to detect specific *SmR2R3-MYB* structural domains. These putative *SmR2R3-MYB* genes were classified based on the phylogenetic grouping of the *Arabidopsis* R2R3-MYB family. The ORF length of these genes ranged from 462 (*EGP15965.1*) to 3051 bp (*EGP09531.1*). The average size of the SmR2R3-MYB proteins was 324.6 aa, ranging from 153 to 1016 aa. The MW of the SmR2R3-MYB proteins ranged from 17.39 (*EGP15965.1*) to 114.3 kDa (*EGP09531.1*). The theoretical pI values ranged from 5 (*EGP19222.1*) to 9.95 (*EGP20989.1*). The predicted GRAVY scores for SmR2R3-MYB proteins ranged from −1.055 (*EGP10787.1* and *EGP17408.1*) to −0.307 (*EGP24348.1*), suggesting that all SmR2R3-MYB proteins were hydrophilic. The amino acid sequences of all genes are presented in Table S1.

### 2.2. Phylogenetic Analysis and Classification of the SmR2R3-MYB Gene Family

**Figure 1:**
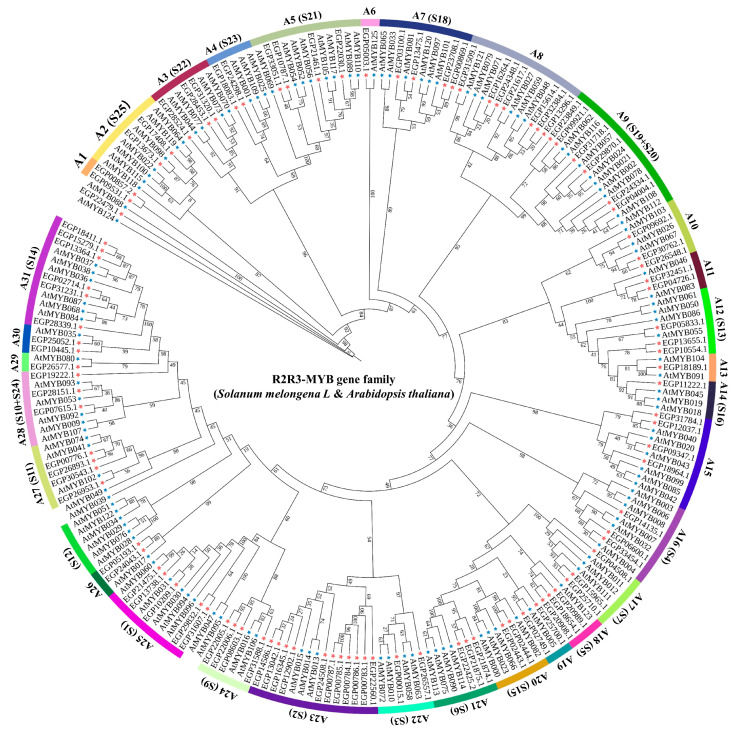
Phylogenetic relationships of eggplant and *Arabidopsis* R2R3-MYB proteins. The ML tree for the complete amino acid sequences of the 126 *Arabidopsis* and 102 eggplant R2R3-MYB were constructed using TBtools with 1000 bootstrap replicates. Blue pentagrams represent *Arabidopsis*; red pentagrams represent eggplants. All R2R3-MYB members were classified into 31 clades (A1~A31).

All SmR2R3-MYB and 126 AtR2R3-MYB proteins were used to construct a phylogenetic tree in order to analyze the phylogenetic relationships and investigate the characteristics of the R2R3-MYBs. TBtools version 2.003 was used to construct the ML phylogenetic tree for the 228 genes, using the *Arabidopsis* R2R3-MYB proteins as a reference. Based on this tree, the *SmR2R3-MYB* genes were classified into 31 subgroups (designated A1 to A31), consistent with the previously reported classification of R2R3-MYBs in *Arabidopsis* [36] as indicated in the evolutionary tree (Figure 1). All eggplant *R2R3-MYB* genes were classified into different subgroups, except for *EPG22479.1*, which was not categorized into any of the groups. Notably, 30 subgroups included different numbers of R2R3-MYB proteins from both species, whereas one subgroup (A1) contained only eggplant members. Most of the eggplant genes belonged to the MYB subfamily of *Arabidopsis* but none were sorted into the *Arabidopsis* S12 subgroup.

### 2.3. Motif, Conserved Domain, and Structure Analyses of SmR2R3-MYBs

**Figure 2:**
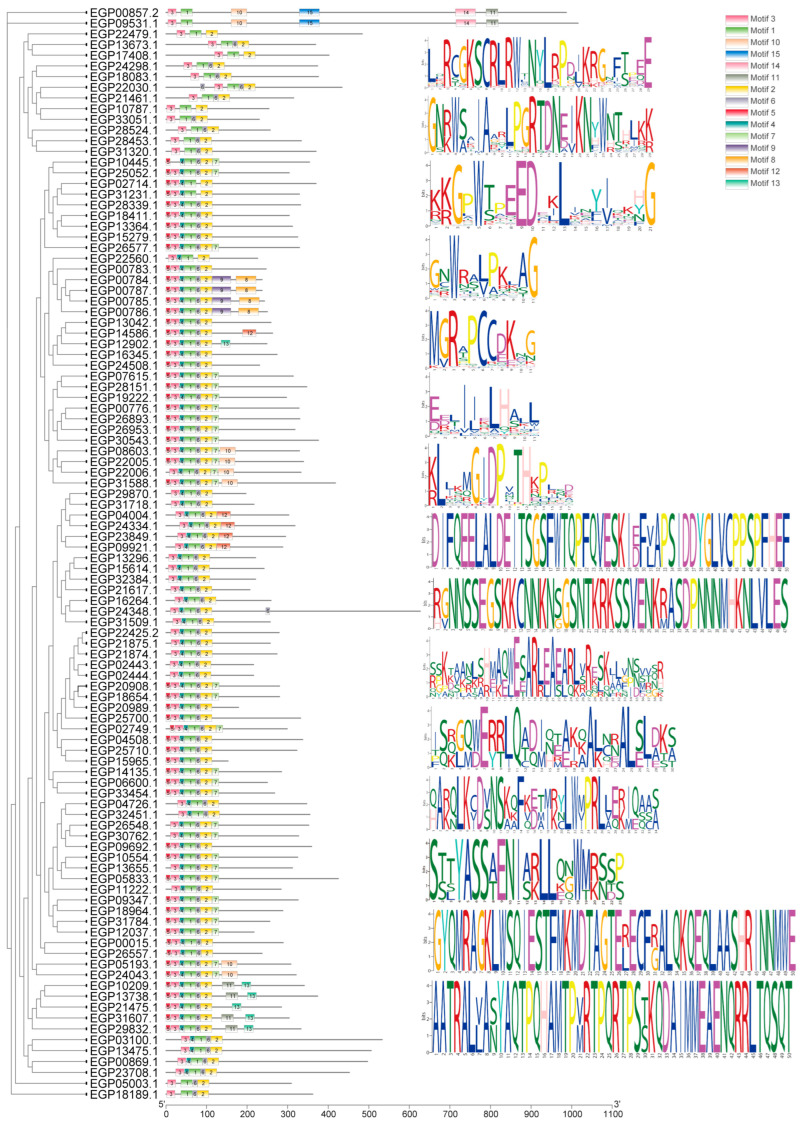
Composition and distribution of conserved motifs of R2R3-MYBs proteins in eggplant fruit. The number of motifs shown in the legend represents the predicted motif groups, the colored boxes represent the different 15 motifs, and the bottom scale indicates the gene lengths.

We used the online MEME program to predict 15 conserved motifs from 102 SmR2R3-MYBs and then visualized the length and conserved sequences of each motif using TBtools software (Figure 2). We found that the composition and distribution of motifs among members within the same subfamily are relatively conserved. Motif 1 and motif 3 are located at the N-terminus of all SmR2R3-MYB protein sequences. The similarity in motif types and numbers within the same subfamily suggests that motif patterns may be related to the function of MYB proteins. Different subfamilies typically have specific motifs; for example, motif 14 and motif 15 are unique to the A1 subfamily, while motif 8 and motif 9 are only present in the A23 subfamily.

**Figure 3:**
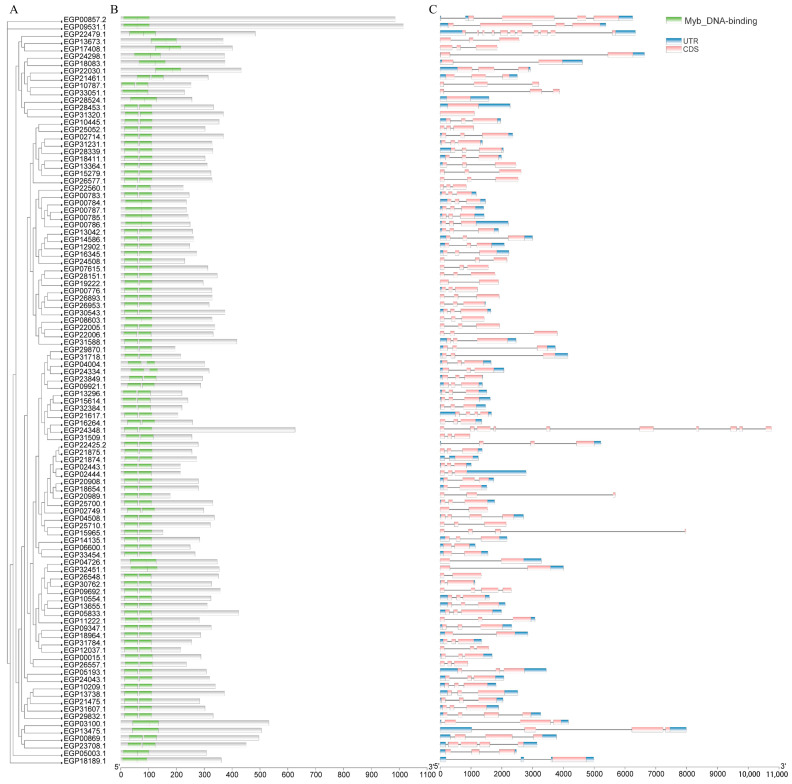
Conserved structural domains and gene structural of R2R3-MYBs in eggplant fruit. (**A**) Phylogenetic tree of 102 R2R3-MYBs proteins. (**B**) Conserved domains of the R2R3-MYBs genes in eggplant fruit. (**C**) Positions of the 3′ noncoding regions (UTR), coding regions (CDS), and intron are indicated by green squares, yellow squares and gray lines.

To further explore the conserved structural domains of SmR2R3-MYB proteins, multiple comparisons of the 102 SmR2R3-MYB protein sequences were conducted using DNAMAN version 6.0.3 The results showed that all SmR2R3-MYB members exhibit the typical features of the MYB-conserved domain (Figure 3B). Structural analysis of the 102 *SmR2R3-MYB* genes revealed that the vast majority of genes (98%, i.e., 100 genes) contain 1~4 coding regions, with 88 *SmR2R3-MYB* genes having 2~3 coding regions. *EGP22479.1* has the highest number of coding regions (11), while three *SmR2R3-MYB* genes lack introns and have only one exon; these genes belong to the A3 subfamily. Most sequence lengths are within 10 kb, with only *EGP24348.1* exceeding 10 kb due to its longer introns (Figure 3C). These findings suggest a high structural similarity within the same subfamily of *SmR2R3-MYB* genes. The conserved features identified through structural analysis may play crucial roles in the specific functions of *SmR2R3-MYB* genes in different subgroups. Although there are differences in the lengths of coding regions and introns, their distribution and quantity indicate a high degree of structural similarity within the same subfamily. Therefore, these conserved features may play key roles in the specific functions of *SmR2R3-MYB* genes in different subgroups.

### 2.4. miRNAs That Target Regulate SmR2R3-MYB Genes

**Figure 4:**
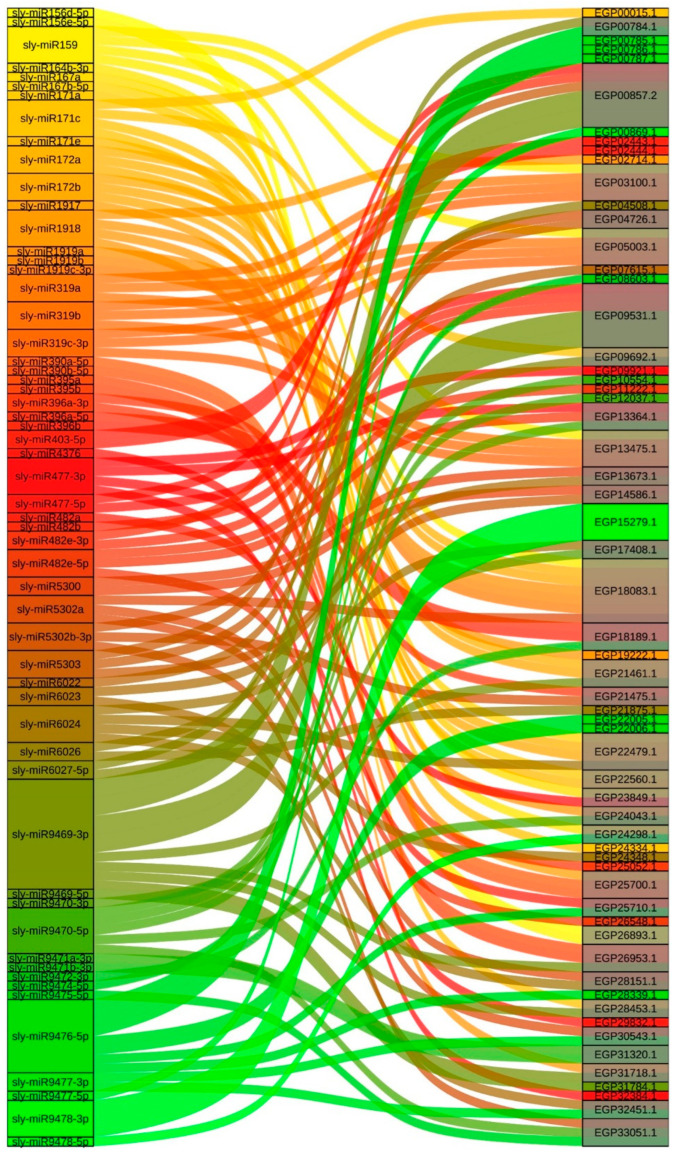
Interaction analysis of potential regulatory network associations between the putative miRNAs and their target SmR2R3-MYB genes. The putative miRNAs are on the left and the target SmR2R3-MYB genes are on the right. The miRNAs and SmR2R3-MYBs connected by different colored lines show potential regulatory associations.

The prediction of miRNAs targeting *SmR2R3-MYB* genes contributes to the exploration of the relationship between plant growth, development, stress responses, and miRNA regulation. The results show that a total of 60 *SmR2R3-MYBs* are targeted by 57 miRNAs. Specifically, three *SmR2R3-MYBs* (*EGP15279.1*, *EGP09531.1*, and *EGP00857.2*) have dual-target sites for specific miRNAs. The miRNA with the highest number of target bindings to *SmR2R3-MYB* genes is sly-miR9476-5p, which has eight target genes, followed by sly-miR9469-3p with six target genes, and sly-miR9470-5p with five target genes. Most other miRNAs target between one and four genes. All other *SmR2R3-MYB* genes have between one and four target sites (Figure 4). These target sites may play crucial roles in the regulation of plant growth, development, and stress responses. The potential miRNA interaction networks of eggplant *R2R3-MYB* genes provide important references for studying their functions and help identify candidate genes for future research.

### 2.5. Chromosomal Distribution and Repeat Events of R2R3-MYB Genes in Eggplant Fruit

**Figure 5:**
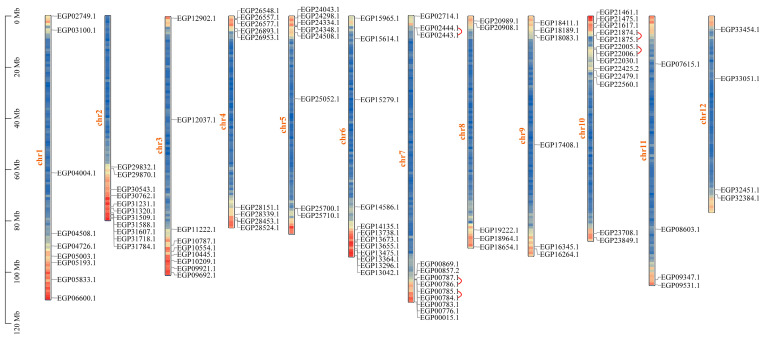
The distribution of eggplant *R2R3-MYB* genes across the chromosomes. The scale bar on the left represents the lengths of the chromosomes (Mb). The red lines represent the duplicated *R2R3-MYB* gene pairs and the chromosome number is indicated in yellow on the left.

The chromosomal data were analyzed using the GFF annotation files to further investigate the chromosomal distribution of the *R2R3-MYB* genes in eggplant fruit. The analysis demonstrated that the 102 identified *SmR2R3-MYB* genes were unevenly distributed on the 12 chromosomes in eggplant fruit, with all chromosomes containing at least four *SmR2R3-MYB* genes (Figure 5). Chromosome 10 (Chr10) contained the largest number of genes (13), while Chr11 and Chr12 had the fewest (4). The remaining chromosomes contained 5 to 12 *SmR2R3-MYB* genes. Gene concatenation and fragment duplication generate gene families during biological evolution [42]. To investigate whether the *SmR2R3-MYB* gene family also underwent replication-based expansion, *SmR2R3-MYB* gene duplication events were investigated. The analysis identified five pairs (9.8%) of tandemly duplicated genes within the *SmR2R3-MYB* genes, distributed on Chr7 and 10 (Figure 5). Furthermore, 26 segmental (25.5%) duplicated pairs were identified among the *SmR2R3-MYB* genes (Figure S1). The largest number of segmental duplications was identified on Chr2 and 6, followed by Chr4. Chr8 contained the lowest number of segmental duplications. The Ka/Ks ratio was calculated for the five pairs of tandemly duplicated and 26 pairs of segmentally duplicated genes (Table S2). The results demonstrated that the Ka/Ks ratio for all gene pairs was less than 1, suggesting that most *SmR2R3-MYB* genes have undergone a negative selection pressure. These findings suggest that some *SmR2R3-MYB* genes may be the result of gene duplication with these duplication events as the most important driving factor in *SmR2R3-MYB* gene evolution.

### 2.6. Comparative Synteny Analysis of R2R3-MYB Genes between Eggplants and Other Species

**Figure 6:**
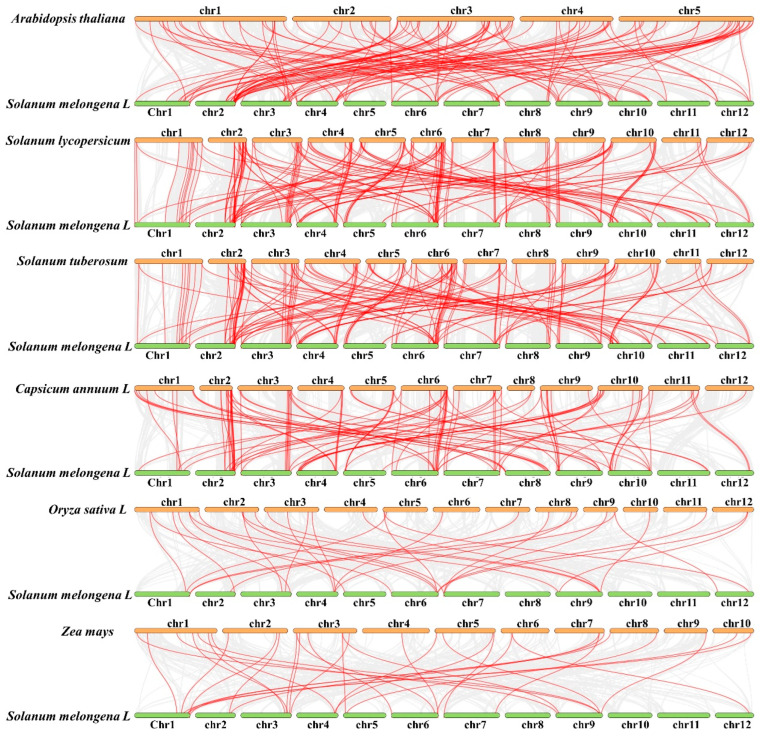
Synteny analysis between eggplants and six representative species. The putative colinear genes between eggplants and six representative species are marked in gray, while the syntenic *R2R3-MYB* gene pairs are marked in red.

To investigate the potential evolutionary processes of SmR2R3-MYB genes, we conducted gene synteny analysis across multiple species to determine the evolutionary relationships between eggplant R2R3-MYB family members and other plant species. This analysis included dicotyledonous plants such as *Arabidopsis*, tomato, potato, and pepper, as well as monocotyledonous plants like rice and maize. The results revealed several syntenic relationships between the 102 SmR2R3-MYB genes and these different plant species. Specifically, there were 64 syntenic gene pairs with *Arabidopsis*, 89 with tomato, 86 with potato, 71 with pepper, 24 with maize, and 20 with rice (Figure 6). It is worth noting that 51 syntenic gene pairs were found between eggplants and *Arabidopsis*, tomato, and pepper, while none were found between eggplants and rice or maize (Table S3). These results suggest that the functions of R2R3-MYB members in eggplant fruit can be inferred from their homologous counterparts in *Arabidopsis* and other Solanaceae crops

### 2.7. Expression Pattern of SmR2R3-MYBs under Diverse Treatments

**Figure 7:**
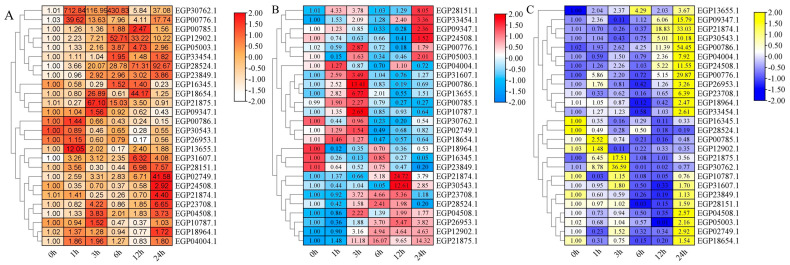
Expression pattern analysis of 26 selected *SmR2R3-MYB* genes in response to treatments based on qRT-PCR results. (**A**) 4 °C. (**B**) ABA. (**C**) PEG. Each column in the heatmap represents a sample and each row represents a gene. Three biological replicates were performed for each sample, with the mean values presented in the heat map. The colors in the graph indicate the normalized expression value of the gene in each sample.

To investigate the functions of SmR2R3-MYB genes in response to environmental stress and hormone treatments, we randomly selected 26 genes and analyzed their expression levels under 4 °C, PEG, and ABA treatments using qRT-PCR. A total of 24 *SmR2R3-MYB* genes exhibited responses to at least one of the treatments, while two (*EGP04508.1* and *EGP33454.1*) did not show any response under all treatment conditions (|log_2_FC| ≥ 2) (Figure 7). Under the treatment of 4° C, a total of 13 genes (*EGP00776.1*, *EGP02749.1*, *EGP05003.1*, *EGP12902.1*, *EGP13655.1*, *EGP18654.1*, *EGP21875.1*, *EGP23708.1*, *EGP28151.1*, *EGP28524.1*, *EGP21874.1*, *EGP30762.1*, and *EGP31607.1*) were upregulated in their transcription levels and 3 genes (*EGP26953.1*, *EGP00786.1* and *EGP16345.1*) showed a downregulation. The expression levels of the other 11 genes did not change significantly.

Among the 16 genes induced by ABA treatment, seven were upregulated (*EGP26953.1*, *EGP12902.1*, *EGP28151.1*, *EGP23708.1*, *EGP13655.1*, *EGP21875.1*, and *EGP21874.1*) and seven genes were downregulated (*EGP16345.1*, *EGP05003.1*, *EGP28524.1*, *EGP00776.1*, *EGP30762.1*, *EGP18964.1*, and *EGP23849.1*). Most of the upregulated genes were activated three hours following treatment, except for *EGP28151.1*, which was activated within one hour of treatment. *EGP05003.1* and *EGP18964.1*, however, were significantly downregulated one hour post-treatment, while others were downregulated after three hours. Under PEG treatment, 24 genes were activated, of which 8 genes (*EGP30543.1*, *EGP24508.1*, *EGP21875.1*, *EGP21874.1*, *EGP13655.1*, *EGP04004.1*, *EGP00786.1*, and *EGP00776.1*) were significantly up-regulated and 11 genes (*EGP28524.1*, *EGP28151.1*, *EGP23849.1*, *EGP18964.1*, *EGP18654.1*, *EGP16345.1*, *EGP12902.1*, *EGP10787.1*, *EGP05003.1*, *EGP02749.1*, and *EGP00785.1*) were significantly down-regulated. Four genes (*EGP33454.1*, *EGP31607.1*, *EGP26953.1*, and *EGP04508.1*) did not respond to PEG treatment. Remarkably, eight genes (*EGP21874.1*, *EGP16345.1*, *EGP05003.1, EGP28524.1*, *EGP00776.1*, *EGP12902.1*, *EGP28151.1*, and *EGP21875.1*) exhibited a consistent response to all three treatments, while two genes (*EGP21874.1* and *EGP21875.1*) demonstrated upregulation in expression across all three experimental conditions.

### 2.8. Generation and Characterization of SmMYB75-OE Transgenic Eggplant Fruit

**Figure 8:**
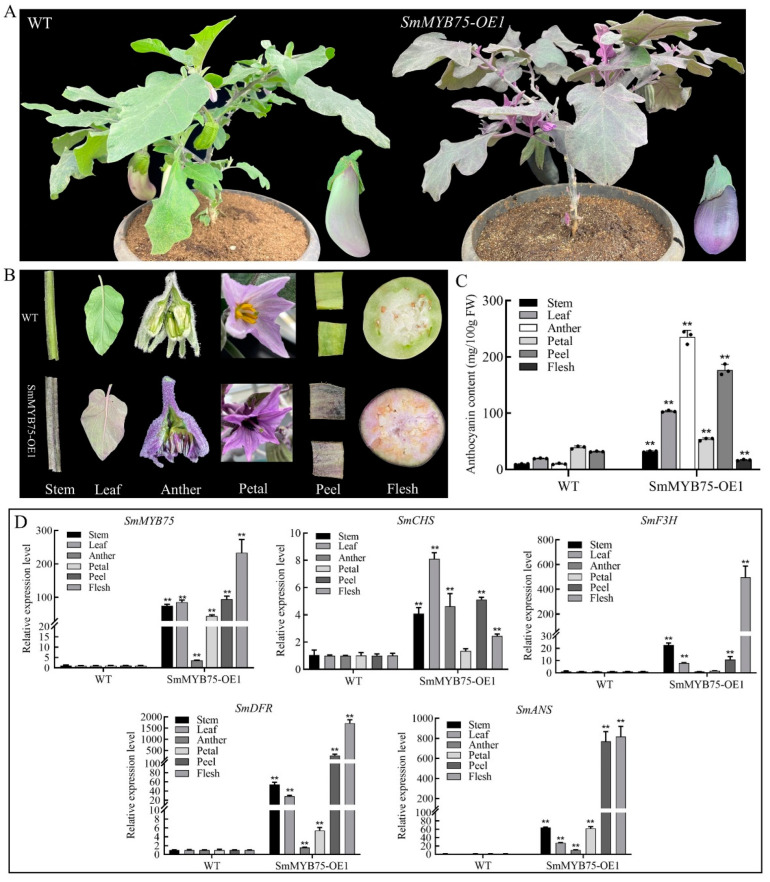
Phenotypic characterization of wild-type and *SmMYB75-OE1* transgenic eggplant plants in different tissues at maturity. (**A**) Overall plant phenotypes. (**B**) Phenotypic characterization of stems, leaves, anthers, petals, pericarp, and flesh in wild-type and *SmMYB75-OE1* plants. (**C**) Comparison of anthocyanin content in six tissues of wild-type and transgenic plants. (**D**) Comparison of relative expression levels of *SmMYB75*, *SmCHS*, *SmF3H*, *SmANS*, and *SmDFR* genes in six tissues of wild-type and transgenic plants. Three biological replicates were used for each sample and the error bars indicate the standard deviation between biological replicates. Asterisks indicates significant differences between groups, ** *p* < 0.01 (*t*-test).

Positive transgenic plants were obtained by overexpressing *SmMYB75* in the eggplant variety ‘LSHX’. Compared with the wild type, the transgenic plants exhibited a distinct purple phenotype overall, with stems, leaves, petals, stamens, pericarp, and pulp turning purple (Figure 8A,B). The anthocyanin content was significantly increased in all tissues (Figure 8C) and the expression levels of *SmMYB75* as well as the structural genes for anthocyanin biosynthesis (*SmCHS*, *SmF3H*, *SmANS*, and *SmDFR*) were significantly up-regulated in all of these tissues (Figure 8D), which further demonstrated the potent function of *SmMYB75* in the biosynthesis of anthocyanins in eggplants.

### 2.9. Anthocyanin-Targeted Metabolomic and Transcriptomic Analysis of Eggplant Flesh

Transcriptome and targeted metabolomic analyses were performed on flesh from *SmMYB75-OE* and WT plants in order to investigate the impact of *SmMYB75* overexpression on anthocyanin synthesis. A total of eight classes of metabolites were detected, including cyanidin, delphinidin, malvidin, pelargonidin, peonidin, petunidin, flavonols, and flavones. Five of the metabolites were upregulated, specifically cyanidin-3-O-(6-O-p-coumaroyl)-glucoside, delphinidin-3-O-glucoside, malvidin-3-O-(6-O-p-coumaroyl)-glucoside, rutin, and kaempferol-3-O-rutinoside (Table S4). Based on the transcriptome data, 2193 DEGs (1404 upregulated and 789 downregulated) were detected between *SmMYB75-OE* and WT (Table S5). KEGG analysis demonstrated that these DEGs were mostly enriched in pathways related to secondary metabolism, flavonoid biosynthesis, and anthocyanin biosynthesis, demonstrating the involvement of *SmMYB75* in anthocyanin biosynthesis (Figure S2A and Table S6). GO classification of these genes was also performed based on biological processes, cellular components, and molecular functions (Figure S2B and Table S7).

**Figure 9:**
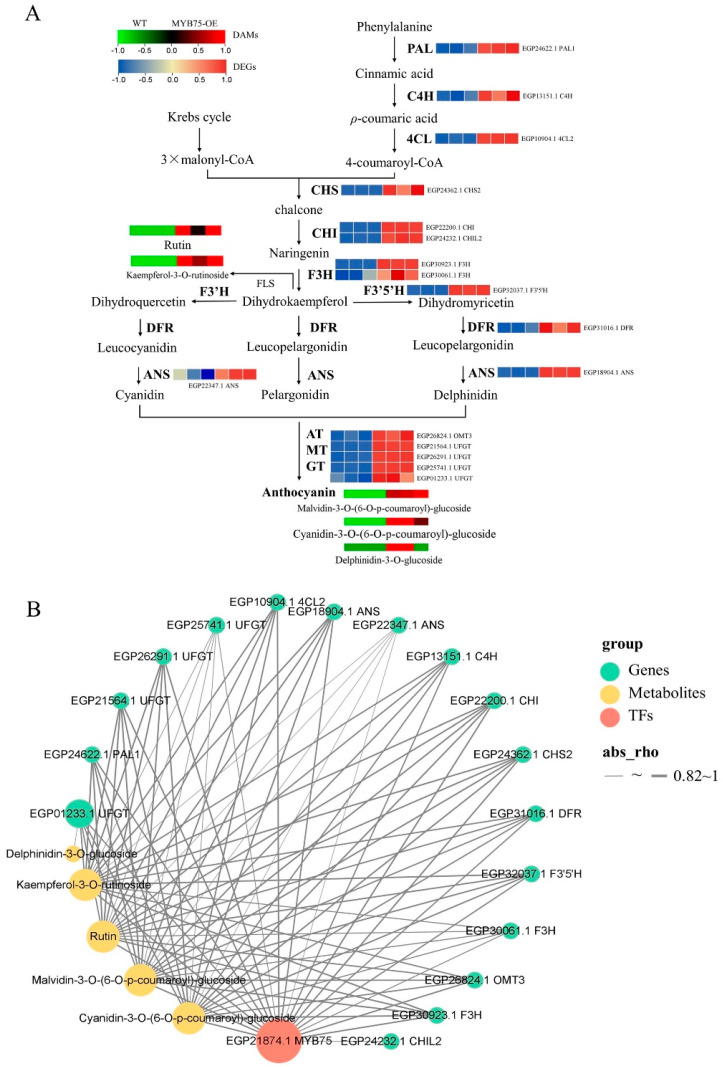
Relationships between structural genes and corresponding metabolites of flavonoid synthesis in the flesh of *SmMYB75* transgenic plants. (**A**) Heatmap of flavonoid synthesis pathways for the upregulated structural genes and DAMs in *SmMYB75-OE* flesh. The green and blue colors represent the downregulation of metabolite accumulation and gene expression, respectively. The dark red and magenta represent the accumulation and upregulation of gene expression, respectively. (**B**) Correlation analysis of *SmMYB75* expression and all DEGs. The content of all DAMs in WT and *SmMYB75-OE* eggplant flesh with correlation coefficients > 0.82 and *p*-values < 0.05, respectively. The size of the plots represents the number of connecting lines.

Based on the biological processes, 18 DEGs were involved in anthocyanin biosynthesis, flavonoid biosynthesis, or metabolic pathways. For example, the genes *SmANS* (*EGP18904.1*), *SmCHI* (*EGP22200.1*), *SmF3H* (*EGP30923.1*), *SmF3′5′H* (*EGP32037.1*), *SmDFR* (*EGP31016.1*), and *Sm3GT* (*EGP21564.1*) were involved in the anthocyanin biosynthetic pathway (ko00942) (Table S7). Furthermore, 12 other DEGs associated with flavonoid biosynthesis were significantly upregulated in *SmMYB75-OE* plants compared to WT (Figure 9A). These results suggest that *SmMYB75* overexpression in eggplants may affect flavonoid (anthocyanin) biosynthesis via structural gene regulation.

The correlation analysis between gene expression and metabolite levels demonstrated a strong positive correlation (correlation coefficient > 0.82) between all DEGs and *SmMYB75*, except for the direct association between delphinidin-3-O-glucoside and *EGP01233* (*3GT*). Furthermore, the other four differentially accumulated metabolites were also directly correlated with *SmMYB75* and multiple other structural genes (Figure 9B). These findings suggest that *SmMYB75* may indirectly affect metabolite accumulation in eggplant fruit flesh via direct regulation of structural genes.

### 2.10. Comprehensive Targeted Metabolic Profiling

**Figure 10:**
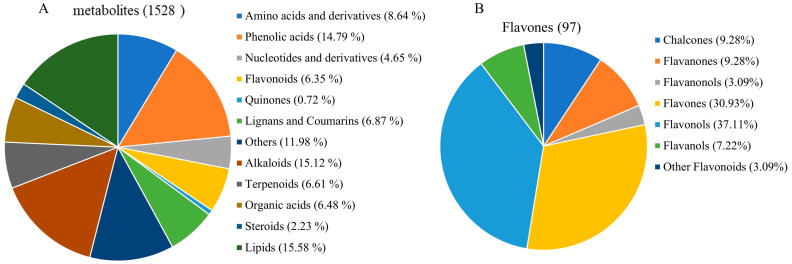
Types and amounts of metabolites detected from WT and *SmMYB75-OE*. (**A**) Classification and percentage of all metabolites. (**B**) Types and percentage of flavonoid metabolites.

To investigate the impact of SmMYB75 overexpression on the metabolic profile of eggplant fruit pulp, a comprehensive targeted metabolomics analysis was conducted by the UPLC-MS platform extensively targeted metabolomic technique. A total of 1528 metabolites were monitored, including 132 amino acids and derivatives, 226 phenolic acids, 71 nucleotides and derivatives, 97 flavonoids, 105 lignans and coumarins, 231 alkaloids, 101 terpenoids, 99 organic acids, 238 lipids, 34 steroids, 11 quinones, and an additional 183 other metabolites (Figure 10A and Table S8). Since anthocyanins are downstream of flavonoid synthesis, we focused on flavonoid metabolites. The 97 flavonoid compounds included 30 flavones, 36 flavonols, 9 chalcones, 9 flavanones, 7 flavanols, 3 flavanonols, and 3 other flavonoids (Figure 10B). PCA analysis showed a significant separation between WT and *SmMYB75-OE*, suggesting that the samples overexpressing *SmMYB75-OE* resulted in significant changes in metabolites in the samples, consistent with the phenotypic changes. Differences in the accumulation patterns of metabolites in different samples could be analyzed by clustering heatmaps and the results showed that there were obvious differences between the two groups of substances and that different biological replicates also clustered together, indicating good homogeneity among the biological replicates and high reliability of the data. From both principal component analysis and cluster analysis, it can be shown that the metabolites produced significant differences between WT and *SmMYB75-OE* (Figure S3).

**Figure 11:**
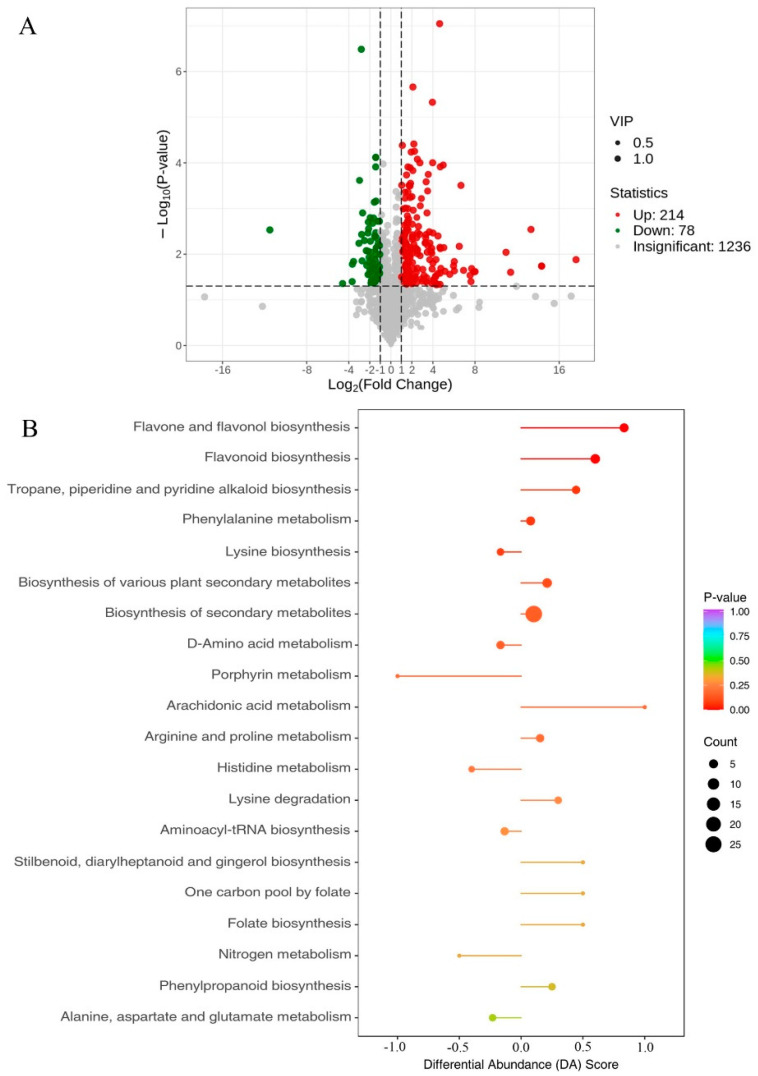
Volcano plot of differential metabolites and KEGG-enriched differential abundance scores. (**A**) Volcano plot. Each point represents a metabolite, where green points represent down-regulated differential metabolites, red points represent up-regulated differential metabolites, and gray points represent metabolites that were detected but did not significantly differ from the horizontal coordinate representative of the logarithm of the multiplicity of the difference in the relative abundance of a metabolite between the two groups of samples (log_2_FC). The larger the absolute value of the horizontal coordinate is, the larger the difference in the relative abundance of the substance between the two groups of samples is. (**B**) Differential abundance score plot. The vertical coordinate indicates the name of the differential pathway (sorted by *p*-value) and the horizontal coordinate indicates the differential abundance score (DA Score). The DA Score reflects the overall changes of all metabolites in the metabolic pathway and a score of 1 indicates that the expression of all identified metabolites in the pathway tends to be up-regulated, while a score of −1 indicates that the expression of all identified metabolites in the pathway tends to be down-regulated.

Fold change and VIP were further utilized to screen for differential metabolites and metabolites had to satisfy both FC > 2, VIP > 1. The results showed that there was a total of 292 differential metabolites in the pulp of wild-type and *SmMYB75-OE* eggplants, of which 214 metabolites were up-regulated and 78 metabolites were down-regulated (Figure 11A, Table S9). All the differential metabolites were matched against KEGG’s database to obtain information on the pathways involved in the metabolites and the annotated results were analyzed by enrichment to obtain the pathways with more differential metabolite enrichment. The results showed that the up-expressed differential metabolites were mainly annotated and enriched in the flavone and flavanol biosynthesis pathway and the flavonoid biosynthesis pathway. The down-regulated differential metabolites were mainly annotated and enriched in the lysine biosynthesis pathway and D-amino acid metabolism pathway (Figure 11B). The flavonoid biosynthesis pathway synthesizes the upstream substances of anthocyanins, which determines the basis of anthocyanin biosynthesis, suggesting that these metabolic pathways were relevant to our study.

## 3. Discussion

The R2R3-MYB gene family is one of the largest in plants, playing various crucial roles in plant physiology [5]. A genome-wide analysis of the R2R3-MYB gene family in eggplants was conducted in order to gain a comprehensive understanding of the gene functions.

A comprehensive query against the eggplant genome was conducted to identify genes encoding *SmR2R3-MYB* transcription factors. Similar to the number of *R2R3-MYB* genes previously found in other plant species such as tomato (133), wolfberry (137), pepper (108), potato (109), and *Arabidopsis* (126) [43], 102 genes were identified in eggplants. The phylogenetic relationships between eggplant fruit and *Arabidopsis* were investigated, aiming to understand the evolution and putative functions of the *SmR2R3-MYBs*. The identified eggplant genes were categorized into 31 subgroups (A1~A31) based on their phylogenetic relationships with the *Arabidopsis* genes. Notably, all *SmR2R3-MYBs* grouped with *AtR2R3-MYBs*, except for *EGP22479.1* (Figure 1), suggesting that the functions of eggplant *MYB* genes can be inferred from their *Arabidopsis* homologs. For instance, *EGP21874.1*, *EGP21875.1*, and *EGP22425.2* clustered with *Arabidopsis AtMYB113, AtMYB114, AtMYB075*, and *AtMYB090* (S6), which are known to be involved in anthocyanin biosynthesis regulation [44]. Similarly, *EGP04508.1*, *EGP15965.1*, and *EGP25710.1* clustered with *AtMYB011, AtMYB012*, and *AtMYB111* (S7) associated with flavonoid accumulation control [45]. Conserved structural domains and gene structure analysis of the eggplant *R2R3-MYB* genes demonstrated that most contained one to six motifs and that genes in the same class contained similar structures (Figure 2). Most *SmR2R3-MYBs* (~80%) contained two or three exons, similar to reports in other plants [46]. Two genes, *EGP22479.1* and *EGP24348.1*, contained more than six introns and multiple non-coding regions that may provide complex structure and function. However, most of the intron and exon sequences were conserved (Figure 3).

An increasing number of studies have shown that miRNAs play key roles in plant growth and development, hormone metabolism, and biotic and abiotic stresses by targeting specific genes. For example, miRNA828-SlMyb7-like inhibits anthocyanin biosynthesis in *Arabidopsis* [47], miRNA858-AtTCPs mediate leaf morphogenesis in *Arabidopsis* [48], and Mdm-miR858 targets *MdMYB9* and *MdMYBPA1* and is involved in anthocyanin biosynthesis in red flesh apple [49]. In the present study, putative miRNAs targeting *SmR2R3-MYBs* were predicted and 60 *SmR2R3-MYBs* were targeted and bound by 57 putative miRNAs. Among these interactions, sly-miR9476-5p exhibited the highest number of bound *SmR2R3-MYB* genes, with eight targets, followed by sly-miR9469-3p with six targets, and sly-miR9470-5p with five targets. Notably, *EGP18083.1* was the *SmR2R3-MYB* gene with the most targets, being targeted by seven miRNAs (Figure 4). These miRNA-SmR2R3-MYB interactions hold the potential to further elucidate their putative roles in eggplant growth, development, and stress responses, contributing to a comprehensive understanding of these processes.

Gene duplication has greatly contributed to the expansion of MYB genes throughout the plant kingdom and is considered to be a major force in gene evolution [15]. Chromosome distribution and gene duplication events analysis identified 10 (9.8%) and 26 (25.5%) *SmR2R3-MYB* genes as tandem and fragmental replications, respectively, suggesting that fragmental replication events are the primary cause of *SmR2R3-MYB* gene amplification. Some evolved members may have lost their original functions or acquired new ones to improve plant adaptation [50]. Tandem and fragmental replication may contribute to the adaptation of eggplant fruit to its environment since the Ka/Ks ratios for five pairs of tandem genes and 26 pairs of fragmental replication genes were less than 1 (Table S2). These results suggest that purifying selection and functional differentiation may have occurred in these genes.

A plethora of studies have demonstrated that R2R3-MYB TFs play a regulatory role in plant-specific processes, including responses to various stresses [51] and secondary metabolic processes [52]. For example, AtMYB41 in *Arabidopsis* is induced when plants are exposed to high salt, abscisic acid (ABA), drought, and cold [53]. Twenty-six SmR2R3-MYB genes were randomly screened and used to analyze the expression under different treatments (PEG, ABA, and 4 °C). A total of 24 genes were responsive to at least one treatment (Figure 7). Among all the genes that were activated or repressed, 12 responded to all three treatment conditions concurrently. This may be because the promoter regulatory elements are stress-responsive. Exceptionally, two genes (*EGP21874.1* and *EGP21875.1*) were up-regulated in expression in all three treatments, suggesting that these two genes may have a strong role in response to stress. Coincidentally, these two genes were also previously reported to promote anthocyanin biosynthesis in eggplant fruit [41,54]. The *SmR2R3-MYBs* exhibited various transcriptional profiles (i.e., activation or repression), suggesting that they have different physiological functions and, specifically, further functional evaluation of *R2R3-MYBs* in eggplant fruit is necessary.

Previous studies have demonstrated that the overexpression of SmMYB75 promotes anthocyanin synthesis in eggplant callus [41]. However, the detailed mechanism of action in eggplant fruit for SmMYB75 and its effects on other metabolites have not been investigated. This study examined the role of this gene in greater detail. Higher expression of *SmMYB75* was correlated with a more pronounced purple color and a corresponding increase in anthocyanin content. The *SmMYB75* gene had a significant impact on anthocyanin biosynthesis in all tissues, including the stem, leaf, petal, anther, pericarp, and flesh in mature eggplants (Figure 8). The flesh, being the edible part of the eggplant, holds substantial nutritional and sensory value. To explore the potential mechanisms of SmMYB75 in eggplant flesh, we conducted transcriptome and metabolome sequencing.

The anthocyanin synthesis pathway in plants is a branch of the flavonoid pathway that is synthesized on the endoplasmic reticulum and, following modification, is transported to the vesicles for storage [55]. Anthocyanin biosynthesis has been relatively well-studied in eggplants but the same cannot be said for the biosynthesis of non-anthocyanin flavonoids [34,56,57]. Transcriptomic data demonstrated significant upregulation of 17 structural genes involved in flavonoid biosynthesis, including *PAL* (*EGP24622.1*), *C4H* (*EGP13151.1*), and *4CL* (*EGP10904.1*), which belong to the phenylalanine synthesis pathway (Figure 9). Anthocyanin-targeted metabolome results suggested that overexpression of *SmMYB75* not only affects anthocyanin biosynthesis but also has a significant consequence on genes of the entire phenylalanine synthesis pathway. In this context, SmMYB75 overexpression led to differential accumulation of three anthocyanins (malvidin-3-O-(6-O-p-coumaroyl)-glucoside, delphinidin-3-O-glucoside, and cyanidin-3-O-(6-O-p-coumaroyl)-glucoside) and two flavonoids (kaempferol-3-O-rutinoside and rutin) in the flesh. Co-analysis revealed that these five metabolites and all differentially expressed genes were highly correlated with SmMYB75 expression levels (Figure 9).

The purple appearance of tea has been previously attributed to a variety of factors, including anthocyanin structure, the pH value surrounding the encapsulated anthocyanin vesicles, and the presence of flavonoids as co-pigments [58]. Therefore, the purple phenotype of eggplant flesh may depend primarily on the accumulation of delphinidin-3-O-glucoside and cyanidin-3-O-(6-O-p-coumaroyl)-glucoside, with two other flavonoids playing a supporting role. This result was similar to the eggplant pericarp, where delphinidin 3-O-glucoside and delphinidin 3-O-rutinoside are also considered key metabolites that determine the degree of the purple color of the eggplant pericarp [54]. However, the mechanism of the intrinsic interaction between the two metabolites requires further investigation. In order to further investigate the impact of the SmMYB75 gene on eggplant fruit quality, we conducted a comprehensive targeted metabolomic analysis of eggplant pulp to identify differential metabolites. Overexpressing SmMYB75 can increase the content of various beneficial compounds such as flavonoids, phenolic acids, and terpenes in eggplant pulp (Figure 10). These compounds have significant effects on plant growth, health, and human nutrition. Anthocyanins, flavonoids, phenolic acids, and terpenes are secondary metabolites found in plants and possess various biological activities, including antioxidant and anti-inflammatory properties. Previously, single overexpression of *SlMYB75* has also been shown to increase the total phenolics, flavonoids, and soluble solids content in tomato fruits. Additionally, it can elevate the content of aroma compounds such as aldehydes, phenylpropanoid-derived substances, and terpene volatiles. These findings indicate that this gene can serve as a key regulatory factor for fruit quality attributes, opening up new avenues for engineering foods with enhanced sensory and nutritional qualities [28]. Therefore, by regulating the SmMYB75 gene, it is possible to enhance the levels of these compounds in eggplants, improving their nutritional value and antioxidant capacity, which is beneficial for human health. This genetic improvement approach can be applied in agriculture to enhance the quality and nutritional value of eggplants.

## 4. Materials and Methods

### 4.1. Identification and Classification of R2R3-MYB Genes in Eggplants

The genome sequences of *Arabidopsis thaliana* and eggplants (*Solanum melongena* L.) were downloaded from The *Arabidopsis* Information Resource (TAIR; https://www.Arabidopsis.org/, accessed on 6 May 2023) and the Eggplant Genome Database (https://db.cngb.org/search/project/CNP0000734/, accessed on 6 May 2023) [59], respectively, to identify R2R3-MYB family members. The previously reported AtMYB protein sequence was used as a query to retrieve the putative eggplant *R2R3-MYB* gene using a two-blast method with TBtools software [60]. The SMART (http://smart.embl-heidelberg.de/, accessed on 7 May 2023) [61] and CDD (https://www.ncbi.nlm.nih.gov/Structure/cdd/cdd.shtml, accessed on 7 May 2023) databases [62] were used for R2R3-MYB structural domain validation of candidate-specific proteins. Finally, 102 eggplant *R2R3-MYB* genes were identified from the eggplant genome. Physicochemical properties of SmR2R3-MYB proteins including isoelectric point (pI), molecular weight (MW), amino acid number (aa), and open reading frame (ORF) were predicted using ExPASy (https://www.expasy.org/, accessed on 7 May 2023).

### 4.2. Phylogenetic Tree Analysis and Classification of R2R3-MYB Genes in Eggplants

An unrooted phylogenetic tree including eggplant and *Arabidopsis* R2R3-MYB proteins was constructed using TBtools software in order to determine the evolutionary relationships between the *R2R3-MYB* genes. First, the ML method was utilized to compare all protein sequences with default parameters with 1000 bootstrap reiterations. The resulting phylogenetic tree of the *SmMYB* genes was then further refined using the interactive tree of life website(iTOL, https://itol.embl.de/, accessed on 10 May 2023). Based on the phylogenetic relationships between eggplant *SmMYB* and *Arabidopsis AtMYB* members, the *SmMYB* gene family has been classified accordingly [63].

### 4.3. Gene Structure, Conserved Domain, and Motif Analysis

The structure and composition of eggplant *R2R3-MYB* genes were examined using the TBtools software. The conserved domains of the proteins were obtained via the Batch CD-Search Tool online. The Multiple Expectation Maximization for Motif Elicitation (MEME) software version 5.0.5 (http://meme-suite.org/tools/meme, accessed on 12 May 2023) was used to identify conserved motifs within the eggplant *R2R3-MYB* genes.

### 4.4. miRNA-R2R3-MYBs Prediction

To forecast the putative miRNA target sites in SmR2R3-MYB genes, we employed all CDS sequences of SmR2R3-MYBs as candidate targets for predicting putative miRNAs. Subsequently, we queried against the candidate targets using the available Solanaceae miRNA mature sequences acquired from the miRbase database (http://www.mirbase.org/, accessed on 13 May 2023) via the psRNATarget server with default parameters [64]. The Sankey plot was employed to present the associations of the putative miRNAs and the corresponding target genes through the Tutools platform (https://www.genedenovo.com, accessed on 13 May 2023).

### 4.5. Chromosomal Localization, Gene Duplication, and Syntenic Analysis

The chromosomal locations and visualization of all SmR2R3-MYB genes were performed using TBtools software. Gene duplication events in eggplants were manually analyzed and represented on the physical map using Multiple Collinear Scanning toolkits (MCScanX) [65]. Genome sequences and gff3 files for *Solanum lycopersicum*, *Solanum tuberosum*, *Capsicum annuum*, *Oryza sativa*, and *Zea mays* were downloaded from Ensembl Plants (http://plants.ensembl.org/index.html, accessed on 13 May 2023). The syntenic relationships between the SmR2R3-MYB genes and these five species were determined using TBtools [60]. Circos was used to visualize segmented replicated gene pairs [66]. Finally, Ka/Ks ratios were calculated using TBtools.

### 4.6. Expression Profiles of Abiotic Stress-Responsive Results and qRT-PCR

The eggplant cultivar ‘LSHX’ was used to characterize the expression patterns of *SmR2R3-MYB* genes in response to phytohormonal and environmental stress conditions. Plants were cultured in a growth chamber with diurnal temperatures ranging from 22 to 25 °C and a 16 h light/8 h dark photoperiod. At the fourth true leaf stage, eggplant seedlings were treated with 100 g/L PEG6000, 100 μM ABA, and low temperature (4 °C). Leaves of three different plants were collected at 0, 0.5, 1, 3, 6, 12, and 24 h post-treatment. Each experiment was performed at least in triplicate. All samples were frozen in liquid nitrogen and stored at −80 °C for future use. Total RNA was isolated using the SteadyPure Plant RNA Extraction Kit (Accurate, Changsha, China) according to the manufacturer’s instructions. First-strand complementary DNA was produced using the Evo M-MLV RT Kit with clean gDNA (Accurate, Changsha, China). The *SmActin* (GU984779.1) gene was used as the internal reference. qRT-PCR-specific primers were designed based on the *SmR2R3-MYB* sequences using the Primer 5 software (Table S1). qRT-PCR was performed using the SYBR^®^ Green Premix Pro Taq HS qPCR kit (Accurate, Changsha, China). Relative mRNA expression was calculated using the 2^−ΔΔCt^ method [67].

### 4.7. Vector Construction and Plant Transformation

The full-length *SmMYB75* CDS without a stop codon was fused to a modified PHB plant expression vector driven by a CaMV35S promoter containing a yellow fluorescent protein (YFP) reporter gene to produce the fusion vector PHB-SmMYB75-YFP. The fusion vector was then transferred into *Agrobacterium tumefaciens* GV3101 using the heat shock method. Eggplant (*Solanum melongena* L.) ‘LSHX’ cultivar was used to generate transgenic plants via *Agrobacterium*-mediated transformation according to a previously reported protocol [68].

### 4.8. RNA-Seq Analysis

Three biological replicates from the eggplant flesh of wild type (WT) ‘LSHX’ cultivar and *SmMYB75-OE* (over-expression) line were collected. The total RNA of the samples was extracted using the RNAprep Pure Plant Kit (DP441, Tianen, China). RNA sequencing (RNA-seq) was performed on the Illumina machine by Metware Biotechnology Co. (Wuhan, China). Clean data were obtained following filtering. The clean reads were then mapped to the reference eggplant genome using HISAT2. The FPKM (fragments per kilobase of transcript per million fragments mapped) method was used to determine the levels of gene expression [69]. Differentially expressed genes (DEGs) between two-sample comparisons were analyzed using the DESeq2 package (version 1.20.0) by applying a significance threshold of *p*-value < 0.05 and |log2 foldchange| ≥ 1. The GO and KEGG analyses were performed following the methodology previously described by Liu et al. [70].

### 4.9. Extraction and Measurement of Total Anthocyanins

The total anthocyanin content in 0.5 g of eggplant samples was measured by pH difference spectrophotometry as previously described [71]. The anthocyanin content was calculated as follows: TA = A × MW × 5 × 100 × V/e; where TA represented the total anthocyanin content (mg/100 g), V was the final volume (mL), and A = [A510 nm (pH 1.0) − A700 nm (pH 1.0)] − [A510 nm (pH 4.5) − A700 nm (pH 4.5)]. The molar absorptivity (e) was 26,900 and the molecular weight (MW) was 449.2. Each sample was performed in triplicate.

### 4.10. Metabolite Analysis

The identification and quantification of anthocyanin metabolites in eggplant pulp samples were determined by a liquid chromatography-electrospray ionization-tandem mass spectrometry (LC-ESI-MS/MS) system from MetWare (Wuhan, China), with detailed descriptions referring to previous studies [72]. Metabolite data analysis was performed with Analyst 1.6.1 software (AB SCIEX, Ottawa, ON, Canada). Supervised multivariate methods, namely partial least squares-discriminant analysis (PLS-DA), were used to maximize metabolome differences between pairs of samples. The relative importance of each metabolite to the PLS-DA model was examined using a parameter called variable importance in projection (VIP). Metabolites with VIP ≥ 1 and fold change ≥ 2 or fold change ≤ 0.5 were considered differential metabolites for population identification [73]. 

### 4.11. Statistical Analysis

All experiments were repeated at least in triplicate and one-way analysis of variance (ANOVA) was performed using IBM SPSS statistical software 20.0 (SPSS Inc., New York, NY, USA) for statistical significance analysis. Differential expression analysis of the RNA-seq data was performed for both WT and OE samples using the DEGseq R package. A *p*-value < 0.05 and |log2(foldchange)| ≥ 1 were set as the threshold for significant differential expression.

## Data Availability

Data is contained within the article and Supplementary Material.

## Supplementary Materials

The following supporting information can be downloaded at https://www.mdpi.com/article/10.3390/ijms25021163/s1.
