# Supplementary material for: Genome-Wide Analysis of R2R3-MYB Genes and Functional Characterization of SmMYB75 in Eggplant Fruit Implications for Crop Improvement and Nutritional Enhancement
Source: Int J Mol Sci. 2024 Jan 18;25(2):1163. doi: 10.3390/ijms25021163 (PMC10816229; doi:10.3390/ijms25021163)
Supplement: Supplementary file 1 [file ijms-25-01163-s001.zip › Figure S1.pdf]

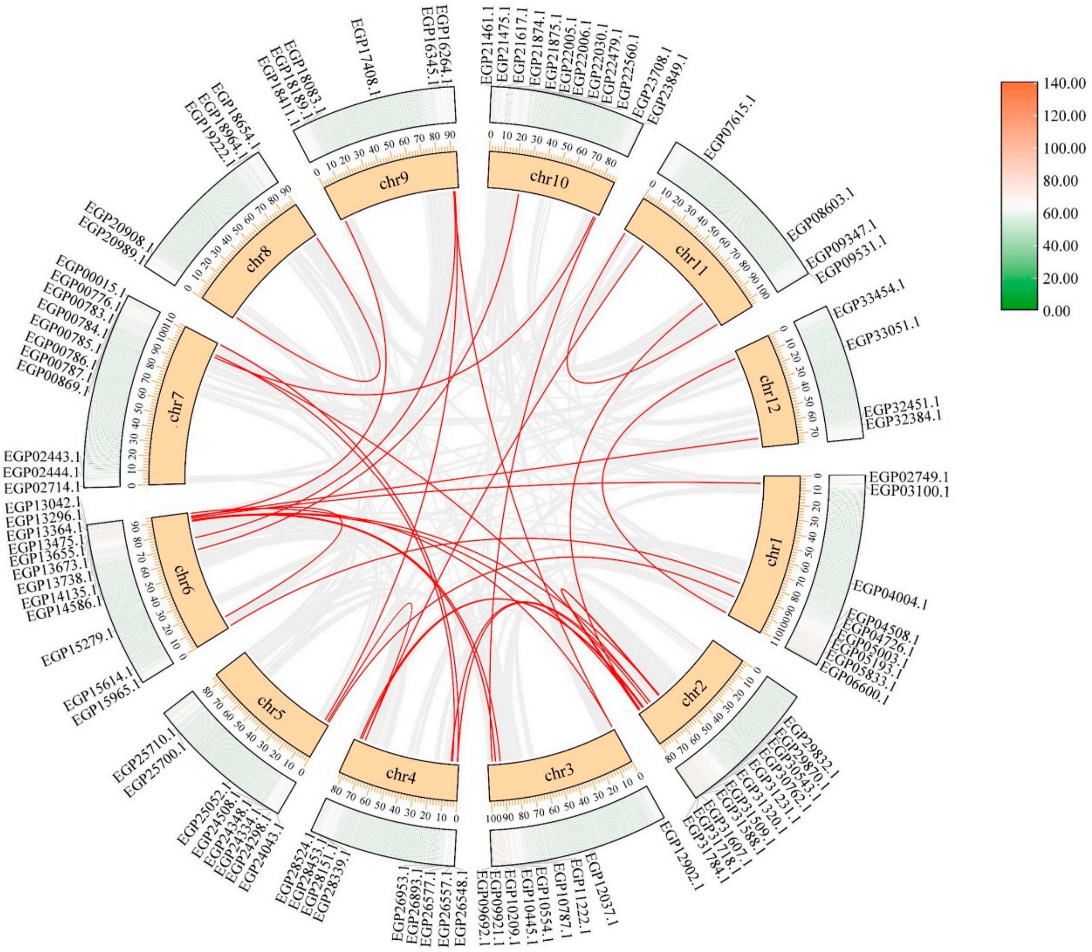

Figure. S1. Schematic representation of the inter-chromosomal correlation between the SmR2R3-MYB genes in eggplant. The red lines represent synthetic blocks in the eggplant genome. The outer circle represents the distribution of gene abundance on different chromosomes
