# Supplementary material for: Genome-Wide Analysis of R2R3-MYB Genes and Functional Characterization of SmMYB75 in Eggplant Fruit Implications for Crop Improvement and Nutritional Enhancement
Source: Int J Mol Sci. 2024 Jan 18;25(2):1163. doi: 10.3390/ijms25021163 (PMC10816229; doi:10.3390/ijms25021163)
Supplement: Supplementary file 1 [file ijms-25-01163-s001.zip › Figure S2.pdf]

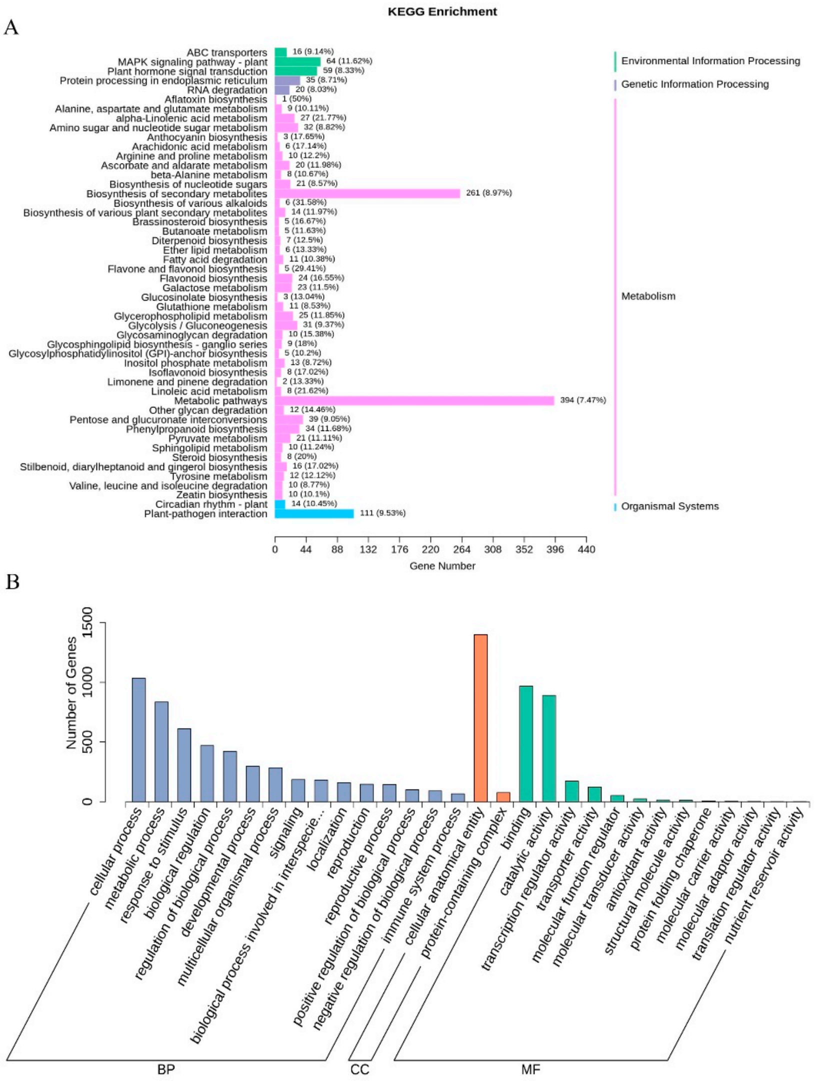

Figure. S2. KEGG and GO analysis of DEGs in the transcriptome. A. KEGG enrichment analysis of DEGs. The horizontal coordinate indicates the number of genes enriched in the pathway. B. GO classification of DEGs identified in the pairwise comparisons. GO terms were sorted based on P-adjust < 0.05
