# Supplementary material for: Genome-Wide Analysis of R2R3-MYB Genes and Functional Characterization of SmMYB75 in Eggplant Fruit Implications for Crop Improvement and Nutritional Enhancement
Source: Int J Mol Sci. 2024 Jan 18;25(2):1163. doi: 10.3390/ijms25021163 (PMC10816229; doi:10.3390/ijms25021163)
Supplement: Supplementary file 1 [file ijms-25-01163-s001.zip › Figure S3.pdf]

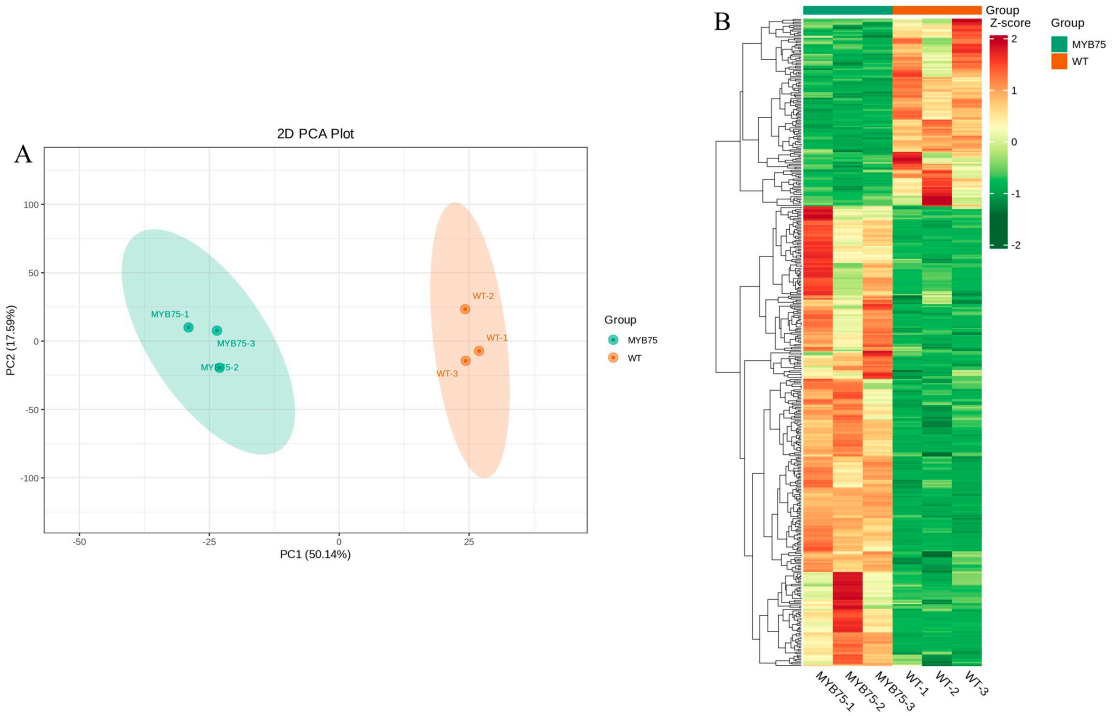

Figure. S3. PCA and Heat Map analysis of relative differential metabolites between SmMYB75 overexpression and wild-type eggplant fruit pulp. (A) PCA score plot. (B) Heat map visualization. Each sample is visualized in separate columns, and each metabolite is represented by individual rows. Red indicates high abundance, and green indicates low abundance.
